# Serum Neurofilament Light Levels in Patients with Cerebrotendinous Xanthomatosis: a Pilot Study

**DOI:** 10.1007/s12311-026-01993-5

**Published:** 2026-04-17

**Authors:** Antonio Edvan Camelo-Filho, Pedro Lucas Grangeiro Sá Barreto Lima, Rodrigo Fagundes da Rosa, Oliver R Miyajima, Matheus Henrique Brustolim Marcucci, Stephanie Suzanne de Oliveira Scott, Luis Edmundo Teixeira de Arruda Furtado, Annyta F Frota, Michelle V R Soares, Danielle S Macedo, André Pessoa, Paulo R Nóbrega, Pedro Braga-Neto

**Affiliations:** 1 Division of Neurology, Department of Clinical Medicine, Universidade Federal do Ceará, Fortaleza, Ceará Brazil; 2 Albert Sabin Hospital, Fortaleza, Ceará Brazil; 3 Center of Health Sciences, Universidade Estadual do Ceará, Fortaleza, Ceará Brazil; 4 Neurology Department, Centro Universitário UNINTA, Sobral, Ceará Brazil; 5 Neuropsychopharmacology Laboratory, Drug Research and Development Center, Department of Physiology and Pharmacology, Faculty of Medicine, Universidade Federal do Ceará, Ceará, Brazil

**Keywords:** Cerebrotendinous xanthomatosis, Ataxia, Nfl, Hereditary ataxia, Autosomal recessive ataxias

## Abstract

Introduction: Cerebrotendinous xanthomatosis (CTX) is a rare autosomal recessive disorder caused by pathogenic variants in *CYP27A1*, leading to cholestanol accumulation and progressive neurological dysfunction. Neurofilament light chain (NfL) is a biomarker of neuroaxonal injury in several hereditary ataxias, but its role in CTX has not been investigated. We aimed to determine whether serum NfL is elevated in CTX and whether it correlates with clinical severity. Methods: Five genetically confirmed CTX patients and five age-, sex-, and body mass index–matched healthy controls were evaluated. Clinical severity was assessed using the Scale for the Assessment and Rating of Ataxia (SARA). Serum NfL concentrations were measured using single-molecule array technology. Group comparisons were performed using the Mann–Whitney U test, and associations were assessed using Spearman’s correlation coefficient. Results: Serum NfL levels were significantly higher in CTX patients than in controls (median 14.63 pg/mL [interquartile range 5.10–18.17] vs. 2.90 pg/mL [2.52–3.56]; p = 0.008). Within the CTX group, NfL levels showed a strong positive correlation with SARA scores (Spearman's ρ = 0.90, p = 0.037), indicating higher NfL levels in patients with greater neurological impairment. Conclusion: Serum NfL is elevated in CTX and correlates with ataxia severity, supporting its potential relevance as a marker of neuroaxonal injury in this disorder. Larger longitudinal studies are needed to confirm these preliminary findings and clarify the role of NfL in disease monitoring.

## Introduction

Cerebrotendinous xanthomatosis (CTX) is a rare autosomal recessive storage disorder resulting from pathogenic variants in the *CYP27A1* gene, which codes for sterol 27-hydroxylase, a member of the cytochrome P450 enzyme family [1]. This enzyme is essential for converting cholesterol into bile acids, and when its function is impaired, cholestanol levels rise and accumulate in several tissues — especially in tendons, ocular structures, and the peripheral and central nervous systems [2–5].

CTX has been recognized as a significantly underdiagnosed disease, a fact that might be related to a highly heterogeneous clinical presentation with a wide range of symptoms, severity, and age of onset [6, 7]. The clinical presentation includes neonatal jaundice or cholestasis, persistent diarrhea, juvenile cataracts, tendon xanthomas, osteoporosis, coronary heart disease, progressive neuropsychiatric issues such as intellectual disability or dementia, psychiatric symptoms, pyramidal and cerebellar signs, progressive myelopathy, extrapyramidal symptoms, seizures, and peripheral neuropathy [1, 6, 8, 9].

Neurofilament proteins, especially neurofilament light chain (NfL), have gained prominence as sensitive and reliable biomarkers of axonal damage and neurodegeneration [10]. As structural components of the neuronal cytoskeleton, neurofilaments are released into the extracellular space and subsequently into blood and cerebrospinal fluid when axonal integrity is compromised [10, 11]. Their quantification has therefore become an essential tool for detecting neuronal injury, monitoring disease progression, and assessing therapeutic responses across a broad spectrum of neurological conditions [10, 12, 13]. In hereditary ataxias, studies have consistently demonstrated markedly elevated NfL concentrations in affected individuals—including those with spinocerebellar ataxias, Friedreich’s ataxia, ataxia-telangiectasia, and RFC1-related cerebellar ataxia—when compared with healthy controls [10, 11, 13–16].

Despite the growing evidence supporting NfL as a biomarker in ataxias, its measurement in CTX has not yet been investigated. In this pilot study, we aimed to determine whether serum NfL could serve as a biomarker reflecting clinical severity in CTX. To test this hypothesis, we measured serum NfL levels in patients with CTX and compared them with healthy controls. We then assessed the relationship between serum NfL concentrations and clinical severity measures.

## Materials and Methods

### Participants

Five patients with CTX were identified from our specialized outpatient clinic for ataxias and rare neurological diseases. All patients had a genetically confirmed diagnosis of CTX and underwent NfL blood analysis and clinical evaluation using a standardized form. The clinical assessment involved a standardized neurological examination, including the Scale for the Assessment and Rating of Ataxia (SARA) [17]. Five healthy individuals without known neurological or systemic disease were recruited as controls for NfL analysis. Control participants were age-, sex-, and body mass index (BMI)-matched to the CTX group. The study adhered to ethical guidelines and was approved by the Ethics Committee at The Universidade Federal do Ceará under protocol number 6.945.762. in compliance with the Declaration of Helsinki. Written informed consent was obtained from all participants. Genetic variants were classified following the American College of Medical Genetics guidelines.

### NfL

Serum NfL concentrations were measured using the Single Molecule Array (Simoa) technology on the Quanterix SR-X platform (Quanterix, Lexington, MA, USA), following the manufacturer’s instructions. All samples were analyzed in duplicate, and operators were blinded to the clinical diagnosis. Internal quality controls and calibration standards were included on each assay plate.

### Statistics

Statistical analyses were performed using Jamovi, version 2.5 (The Jamovi Project, Sydney, Australia). Continuous variables are presented as mean ± standard deviation or median (interquartile range), as appropriate. Because serum NfL values demonstrated skewed distributions and the CTX sample size was small, nonparametric methods were used for primary analyses. Group differences in serum NfL between CTX patients and controls were assessed using the Mann–Whitney U test. Associations between serum NfL and continuous clinical variables, including SARA score, were evaluated using Spearman’s rank correlation coefficient. Statistical significance was defined as *p* < 0.05.

## Results

This study included five patients with CTX. Table 1 summarizes the clinical profile of patients. The median age at evaluation was 31 years (range: 9–40), and the mean disease duration was 26 years (range: 6–39). Symptoms started mainly in childhood and adolescence, and most patients developed psychiatric and neurological symptoms with significant variation in additional clinical features. Parental consanguinity was reported in three out of five patients. All patients experienced a significant diagnostic delay, with a mean interval of 18.6 years (range: 9–31 years) from symptom onset to diagnosis. Four patients presented with cerebellar ataxia. Three of the five patients are currently receiving chenodeoxycholic acid (CDCA) therapy – the standard treatment for reducing cholestanol accumulation. Other common findings included chronic diarrhea (60%), cataracts (60%), and pyramidal signs (60%). There were no significant differences in sex distribution (*p* = 1.00) or age (*p* = 0.69) between CTX patients and controls (see Table 2).

**Table 1:** Clinical, Demographic, and Genetic Characteristics of Patients with Cerebrotendinous Xanthomatosis

	Case 1	Case 2	Case 3	Case 4	Case 5
Age at evaluation (y)	9	34	27	31	40
Age of diagnosis (y)	6	29	25	31	39
Disease duration	9	16	12	31	25
Gender	F	F	M	F	M
BMI	14.1	25.9	18.3	21.2	26.4
First symptom	Neonatal cholestasis	Tendon Xanthomas	Cataract	Neonatal cholestasis	Tendon Xanthomas
Number of Xanthomas	0	5	2	4	4
SARA	0	2	3	8	25.5
PND	0	0	0	1	3a
Polyneuropathy	-	**-**	-	Demyelinating	Demyelinating
Cholestanol Brain MRI CDCA use	High Abnormal YES	High Abnormal YES	N/A Normal NO	N/A Abnormal NO	High Abnormal YES
Variant 1	c.1183 C > T (p.Arg395Cys)	c.886 C > T (p.Gln296*)	c.1183 C > T (p.Arg395Cys)	c.379 C > T (p.Arg127Trp)	c.1181T > C (p.Leu394Pro)
Variant 2	c.1183 C > T (p.Arg395Cys)	c.1421G > A (p.Arg474Gln)	c.1183 C > T (p.Arg395Cys)	c.379 C > T (p.Arg127Trp)	c.1181T > C (p.Leu394Pro)
Other clinical features	Intellectual disability Psychiatric symptoms Pyramidal Signs Chronic diarrhea	Psychiatric symptoms Pyramidal Signs Chronic diarrhea	Cataracts Psychiatric symptoms	Cataracts Psychiatric symptoms Cognitive decline Chronic diarrhea	Cataracts Psychiatric symptoms Cognitive decline Pyramidal Signs Epilepsy *Pes cavus*

This table summarizes individual patient clinical data. Abbreviations: BMI = Body mass index; SARA = Scale for the Assessment and Rating of Ataxia; PND = Polyneuropathy Disability score; CDCA = Chenodeoxycholic acid; MRI = Magnetic resonance imaging; N/A = Not available

**Table 2:** Demographic and Clinical Characteristics of CTX Patients and Controls

Variable	CTX (*n* = 5)	Controls (*n* = 5)	*p*
Age (y)	31 (9–40)	33 (11–35)	0.69
Female sex, n (%)	2 (40%)	2 (40%)	1.00
Male sex, n (%)	3 (60%)	3 (60%)	
BMI, kg/m²	21.2 kg/m² (IQR 18.3–25.9)	22.77 kg/m² (IQR 21.95–29.72)	0.40
Disease duration, years	16 (9–31)	—	
SARA score	3.0 (0–25.5)	—	
Polyneuropathy, n (%)	2 (40%)	0	
MRI abnormalities, n (%)	4 (80%)	0	
Tendon xanthomas, n (%)	4 (80%)	0	

**Fig. 1:**
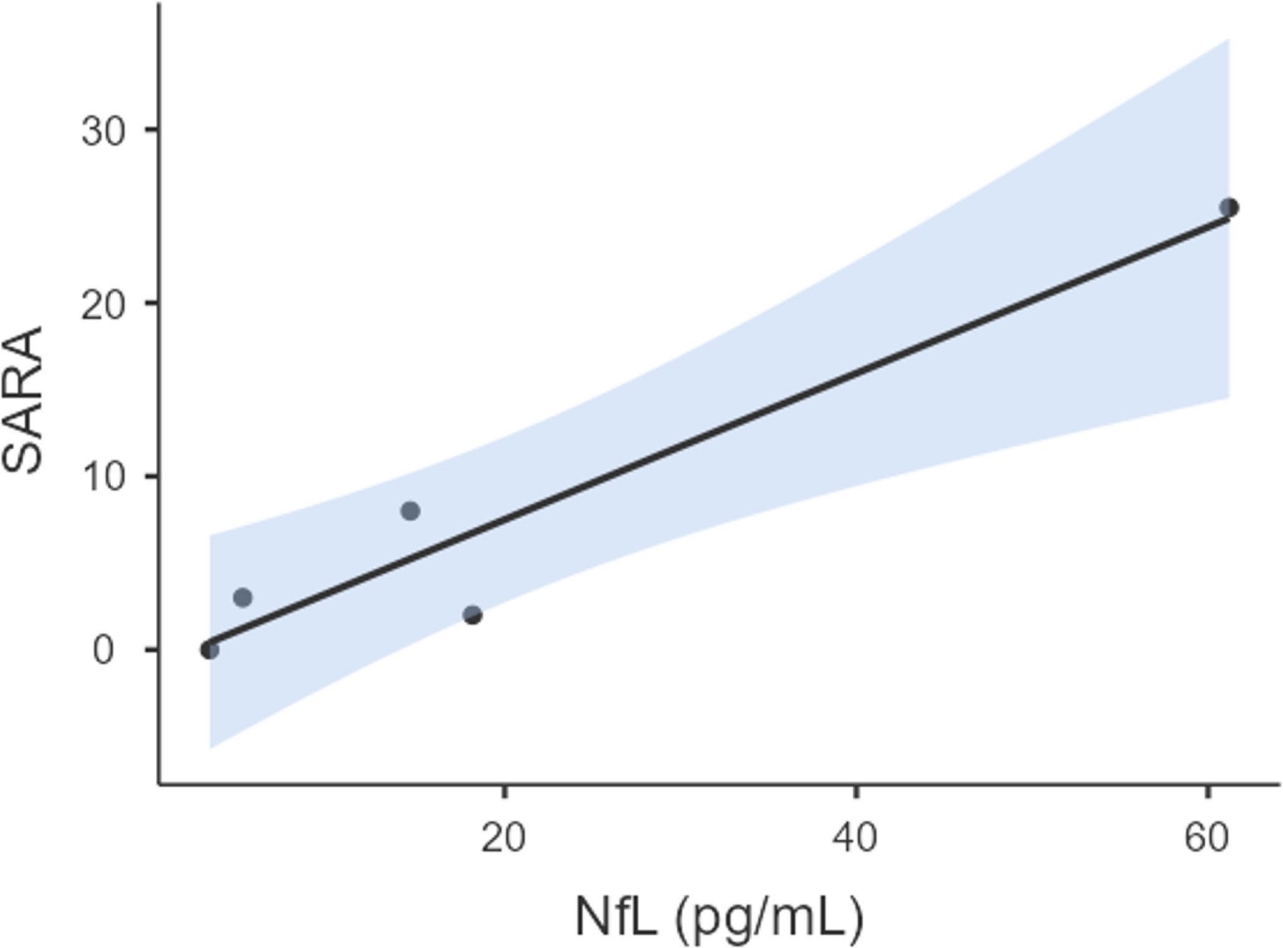
NfL Levels and Ataxia Severity

Serum NfL concentrations were markedly higher in CTX patients compared with healthy controls. The median NfL level in the CTX group was 14.63 pg/mL (IQR 5.10–18.17), whereas the control group had a median of 2.90 pg/mL (IQR 2.52–3.56) (Mann–Whitney U test, *p* = 0.008). Within the CTX group, serum NfL levels showed a statistically significant positive correlation with SARA scores (Spearman ρ = 0.90, *p* = 0.037), indicating that higher neurofilament levels were associated with greater neurological severity (Fig. 1).

A significant positive correlation was observed between NfL concentrations and SARA scores (Spearman ρ = 0.90, *p* = 0.037). Abbreviations: NfL = Neurofilament Light Chain, SARA = Scale for the Assessment and Rating of Ataxia.

## Discussion

This pilot study provides preliminary evidence that serum NfL is elevated in patients with CTX and possibly reflects clinical severity. CTX patients had significantly higher NfL concentrations than healthy controls, and NfL was strongly associated with SARA scores, supporting its potential as a biomarker of neuroaxonal injury in this condition.

The observed effect is consistent with findings in other inherited ataxias, where NfL correlates with disease burden and functional decline [13, 14, 18]. These findings support the idea that serum NfL may capture clinically meaningful aspects of neurological involvement in CTX. If confirmed in larger and longitudinal studies, NfL could help monitor disease progression more objectively and potentially serve as a complementary marker alongside clinical scales such as SARA.

Our study has some limitations. First, this was an exploratory analysis with a small number of participants, which limits the strength of the conclusions and precludes subgroup comparisons or formal validation. Second, a cross-sectional design prevents any assessment of changes in serum NfL over time. Third, three patients were receiving CDCA at the time of blood sampling, which may have influenced serum NfL levels and introduced additional variability into the results. Longitudinal, prospective studies are needed to determine whether NfL can track disease progression or assist in earlier diagnosis.

## Conclusion

In conclusion, our findings indicate that serum NfL may be a marker of neurodegeneration and clinical worsening in CTX. Because it is easy to obtain and measure, serum NfL could help clinicians objectively follow patients over time and possibly assess treatment effects. Nevertheless, these results are preliminary. Studies with larger cohorts and long-term follow-up are still needed to confirm whether serum NfL can be reliably used as a biomarker in CTX.

## Data Availability

All data produced is included in the manuscript.

## Abbreviations

ACMG: American College of Medical Genetics
BMI: Body Mass Index
CDCA: Chenodeoxycholic Acid
CTX: Cerebrotendinous Xanthomatosis
CYP27A1: Cytochrome P450 Family 27 Subfamily A Member 1
IQR: Interquartile Range
MRI: Magnetic Resonance Imaging
NfL: Neurofilament Light Chain
PND: Polyneuropathy Disability score
SARA: Scale for the Assessment and Rating of Ataxia
SCA: Spinocerebellar ataxia
Simoa: Single Molecule Array
